# Assessing Parkinson’s Disease at Scale Using Telephone-Recorded Speech: Insights from the Parkinson’s Voice Initiative

**DOI:** 10.3390/diagnostics11101892

**Published:** 2021-10-14

**Authors:** Siddharth Arora, Athanasios Tsanas

**Affiliations:** 1 Somerville College, University of Oxford, Oxford OX2 6HD, UK; Siddharth.Arora@maths.ox.ac.uk; 2 Usher Institute, Edinburgh Medical School, University of Edinburgh, Edinburgh EH16 4UX, UK

**Keywords:** acoustic measures, biomarker, clinical decision support tool, dysphonia measures, Parkinson’s disease, sustained vowel phonations, telemonitoring

## Abstract

Numerous studies have reported on the high accuracy of using voice tasks for the remote detection and monitoring of Parkinson’s Disease (PD). Most of these studies, however, report findings on a small number of voice recordings, often collected under acoustically controlled conditions, and therefore cannot scale at large without specialized equipment. In this study, we aimed to evaluate the potential of using voice as a population-based PD screening tool in resource-constrained settings. Using the standard telephone network, we processed 11,942 sustained vowel /a/ phonations from a US-English cohort comprising 1078 PD and 5453 control participants. We characterized each phonation using 304 dysphonia measures to quantify a range of vocal impairments. Given that this is a highly unbalanced problem, we used the following strategy: we selected a balanced subset (*n* = 3000 samples) for training and testing using 10-fold cross-validation (CV), and the remaining (unbalanced held-out dataset, *n* = 8942) samples for further model validation. Using robust feature selection methods we selected 27 dysphonia measures to present into a radial-basis-function support vector machine and demonstrated differentiation of PD participants from controls with 67.43% sensitivity and 67.25% specificity. These findings could help pave the way forward toward the development of an inexpensive, remote, and reliable diagnostic support tool for PD using voice as a digital biomarker.

## 1. Introduction

Neurological diseases strain health systems and pose a considerable ongoing burden on healthcare resources. Parkinson’s Disease (PD) has been reported as one of the fastest-growing neurological disorders in terms of prevalence and deaths [1]. A large, global burden of disease study identified PD as one of the top 5 leading causes of death from neurological disorders in the US [2]. It is estimated that there were approximately 6.1 million people with PD (PwP) globally in 2016, indicating a sharp upward trend compared to 2.5 million PwP in 1990 [1]. 

Diagnosis of PD requires subjective assessment in-clinic, which incurs logistical costs. Crucially, consultant neurologists might misdiagnose PD in up to around 20% of the total cases, while the symptom monitoring accuracy is inherently limited due to the intra- and inter-rater variations in the standard clinical scales used to assess PD symptoms’ severity [3,4]. Given the current objective constraints and limitations with subjective assessments, there is an urgent and unmet need for developing diagnostic support tools for the objective detection and monitoring of PD.

Parkinson’s disease is a neurodegenerative disease that is characterized by four cardinal signs: tremor, bradykinesia, rigor, and postural instability [5]. Most PwP also experience some form of speech performance degradation as a result of PD [6]. It is due to this reason, that the potential of capitalizing on acoustic analysis of speech signals to develop PD decision support tools has been pursued vigorously with considerable success over the last 10–15 years. Encouragingly, using voice, studies have proposed technologies based on acoustic analyses to: (1) differentiate PwP from controls [7,8,9,10], (2) monitor the symptom severity of PD [11,12,13,14], (3) assess voice rehabilitation in PD [15], (4) identify at-risk participants (i.e., those with isolated Rapid Eye Movement (REM) sleep behavior disorder as confirmed by a polysomnography test) [16], (4) identify participants with a higher genetic predisposition for developing PD (i.e., those with a mutation in the Leucine-Rich Repeat Kinase 2 (LRRK2) gene) [17], and (5) predict a range of clinical scores that quantify participants’ motor symptoms, cognition, daytime sleepiness, depression, and overall state of health [18]. A limitation of these studies was, however, that they typically rely on using high-quality voice recordings for the analyses which are collected under carefully acoustically controlled conditions with high-end specialized equipment. 

Recently, to assess the scalability of voice as a population screening tool for PD, we undertook the largest PD characterization study employing telephone-quality voice [19], which we refer to as the Parkinson’s Voice Initiative (PVI) study. PVI is the first of its kind large-scale study collecting speech data from PwP and control participants under free-living acoustic conditions. Using sustained vowel phonations (International Phonetic Alphabet /a:/) collected from participants in 7 countries, Arora et al. [19] sought to discriminate PD participants from controls using phonations collected under non-acoustically controlled conditions 

The use of sustained phonations for quantifying vocal impairment is well established [20,21]. However, our understanding of variations in dysphonia measures/sustained phonations from participants with different linguistic backgrounds is still rather limited. Historically, the use of sustained vowels has been motivated by the fact that they can be considered generic (certain vowels such as /a/ are met across different languages) and hence the processing of sustained vowel phonations overcomes linguistic differences [20]. In their analyses, Arora et al. (2019) [19] relied on the underlying assumption that sustained vowel phonations are considered generalizable across people from different linguistic backgrounds, pooling together all the data from PVI. Tsanas and Arora (2021) [22] investigated the differences in dysphonia measures between UK- and US-English speaking PwP, and reported that although there is an excellent agreement between classical acoustic measures (such as jitter and shimmer), there are pronounced differences in some of the more advanced acoustic measures between the two cohorts. Given that phonations may be language-dependent, this prompts the further question of whether acoustic analyses should be performed separately for participants from different linguistic backgrounds, along with undertaking cross-cohort comparisons. Therefore, this study is a natural extension of the work undertaken by Arora et al. (2019) [19], whereby we focus on the stratified analysis of the sustained phonation by using voice recordings from participants from one linguistic background, specifically, the US-English cohort. 

The paper is organized as follows. Section 2 presents the data, followed by the methodology used for acoustic analysis comprising data pre-processing, feature extraction, feature selection, classification, and evaluation strategy. Section 3 presents the results, focusing on describing the most salient dysphonia measures that differentiate PwP from controls, along with the out-of-sample classification results. Discussions and directions for future research are provided in Section 4. Conclusions are provided in Section 5. 

## 2. Data and Methods

### 2.1. Data Characteristics

We processed sustained vowel (/a/) phonations collected as part of the PVI. The recordings were sampled at 8 kHz with 16 bits resolution and were collected via telephone digital audio lines. The participants were instructed to say ‘aaah’ as steadily and for as long as possible. All calls were non-identifiable, and participants were entirely self-selected. During the call, participants were asked to provide basic demographics (age, gender) and whether they have received a clinical PD diagnosis. For further details on the data collection protocol, please see Arora et al. (2019) [19]. As mentioned previously, here we focus on the cohort where we had the largest participation (US) in the PVI study, and aim to progressively explore further differences in follow-up work.

Table 1 presents the data details and participant characteristics of the US PVI-cohort that is used hereafter. A total of 12,675 phonations from 6942 participants were originally collected. We used an automated algorithm to exclude phonations that had excessive background noise, erroneous recordings, or were otherwise missing information following the methodology we previously described [19,23]. Specifically, 1987 phonations from 1078 PD participants and 9955 phonations from 5453 controls were further processed.

### 2.2. Dysphonia Measures

We acoustically characterized each sustained vowel /a/ phonation using speech signal processing algorithms to extract 304 dysphonia measures. These dysphonia measures have been developed specifically to characterize sustained vowel /a/ phonations in the context of PD voice assessment, quantifying physiological patterns including deviation from vocal fold periodicity (jitter and shimmer variants), acoustic/turbulent noise, and articulator placement. For the rationale, background and detailed algorithmic expressions for the computation of the dysphonia measures, we refer interested readers to our previous work [12,21,24,25]. The MATLAB source code for the computation of the dysphonia measures is freely available from the last author’s website: https://www.darth-group.com/software (last accessed 10 October 2021). For completeness, we succinctly summarize these algorithms in Table 2, categorized in algorithmic families along with a brief description. 

The fundamental frequency (F0) is a critical component in speech signal analysis and is often used as a pre-processing step for many of the dysphonia measures such as jitter [20,21]. Strictly speaking, F0 is only defined for strictly periodic signals. In practical speech signal processing, we use the concept of F0 to refer to the vibrating pattern of the vocal folds in the short term and typically compute the F0 contour in short pre-specific segments (typically every 10 ms) [12,26,27]. This is, therefore, a practically applicable approach even in speech signals which are not periodic [12,26]. Here, we computed F0 using the Sawtooth Waveform Inspired Pitch Estimator (SWIPE) algorithm [28] which we have previously reported is one the most accurate F0 estimators in the context of sustained vowels [29]. We clarify that we processed only the most stationary 2 s signal segment from each phonation, which was determined by identifying the least-fluctuating 2 s continuous F0 contour segment (in 10 ms steps) as determined using SWIPE; this circumvents problems with highly fluctuating signals. Applying the speech signal processing algorithms gave rise to an 11,942 × 304 feature matrix that was subsequently processed to map onto the binary outcome (0 was used to denote controls and 1 to denote PwP).

### 2.3. Dimensionality Reduction Using Feature Selection and Feature Transformation

High dimensional datasets often lead to well-known problems broadly referred to as the curse of dimensionality. In short, the presence of a large number of noisy and redundant features may affect the predictive performance of the statistical learning algorithm [30]. To address this problem, traditional feature selection or feature transformation approaches were used, aiming to reduce the dimensionality of the dataset before presenting it to the statistical learner. We indicatively used three feature selection methods and one feature transformation method to explore different approaches to the problem of optimizing the out-of-sample performance of the subsequent statistical learner. Specifically, we applied the following feature selection methods: (1) GSO [31], (2) RELIEF [32], and SIMBA [33]. Each of these feature selection methods provides a ranking of the features. In each case, we used the feature selection voting strategy we had previously introduced [10,15] to robustly determine the final feature subset for each feature selection algorithm. In all cases, we restricted the search to the top 30 features selected using each algorithm. Finally, we explored feature transformation using standard principal component analysis (we extracted the first 30 principal components).

### 2.4. Statistical Mapping

We have used three state-of-the-art statistical mapping algorithms: (1) Random Forests (RF) [34], (2) Support Vector Machines (SVM) [35], (3) Adaptive Boosting (AdaBoost) [36] to tackle the binary differentiation problem in the study. We chose these methods as they are commonly used off-the-shelf classifiers that have been shown to be accurate in diverse supervised learning problems and, in particular, in a similar context differentiating PwP from controls using voice [18,19,25]. For the RF we explored optimizing performance using Breiman’s recommendation with half and twice the default recommended number of features over which to select features for each node, and explored findings using 500 trees and 1000 trees. For the SVM we used the LIBSVM implementation with a MATLAB wrapper [37] and followed the suggestions of the developers of that implementation for optimizing the hyper-parameters [38]: we linearly scaled each of the features to lie in the range [−1, 1], and used a Gaussian, radial basis function kernel. We clarify that for the scaling of the features in both the training and the testing subsets, only the information from the training subset was used. The penalty parameter *C* and the kernel bandwidth *w* were determined using a standard grid search (*C*, *w*) defined by the product of the sets $C=\left[2^{-5},\;2^{-13},\ldots ,\;2^{15}\right]$, and $w=[2^{-15},2^{-13},\ldots ,\;2^{3}]$. The optimal parameter pair $\left(C,w\right)$ was determined using the highest balanced accuracy. For Adaboost, the learning rate hyper-parameter was optimized in the 0.01 to 0.5 range (we searched the following possible values: 0.01, 0.03, 0.05, 0.1, 0.3, and 0.5) and the number of trees used as weak base learners of the boosted classifier was set to 1000. We refer to the original papers and Hastie et al. [30] for an authoritative description of the methods and further details on parameter fine-tuning and optimization. 

Given that the dataset is highly unbalanced (9955/11,942 samples are from controls and 1987/11,642 samples from PwP, i.e., >80% samples in the dominant class) and this setting is known to be particularly challenging for statistical learning models [39], we wanted to explore a different strategy to mitigate potential problems due to one class dominating the performance of the classifiers. The strategy we followed for training and testing the model comprises two steps. 

In the first step, we randomly selected 1500 samples from PwP and 1500 samples from controls to create a balanced binary classification dataset (*n* = 3000 samples) which we will use to train, explore, optimize, and validate the classifiers. To assess model validation for this balanced dataset we used a standard 10-fold cross-validation with a 100 iterations, following the standard methodology that we had previously used in similar applications in this field [10,12,18]. The aim is to use this first step to decide on the final model, by optimizing and setting any hyper-parameters so that it can be finalized and used externally in new datasets. We clarify that the feature selection and feature transformation approaches were applied using only the balanced dataset. We report performance on the out-of-sample CV data. 

The second step is used as a final model validation assessment where we have used the remaining data that was not already used in step 1. In this case, we have an unbalanced dataset with the remaining samples (8942 samples, 8455 recordings from controls and 487 recordings from PwP). This is used to provide further evidence of the model generalization performance with samples that have not been used for any of the preceding steps with feature selection/transformation and statistical mapping. 

**Figure 1:**
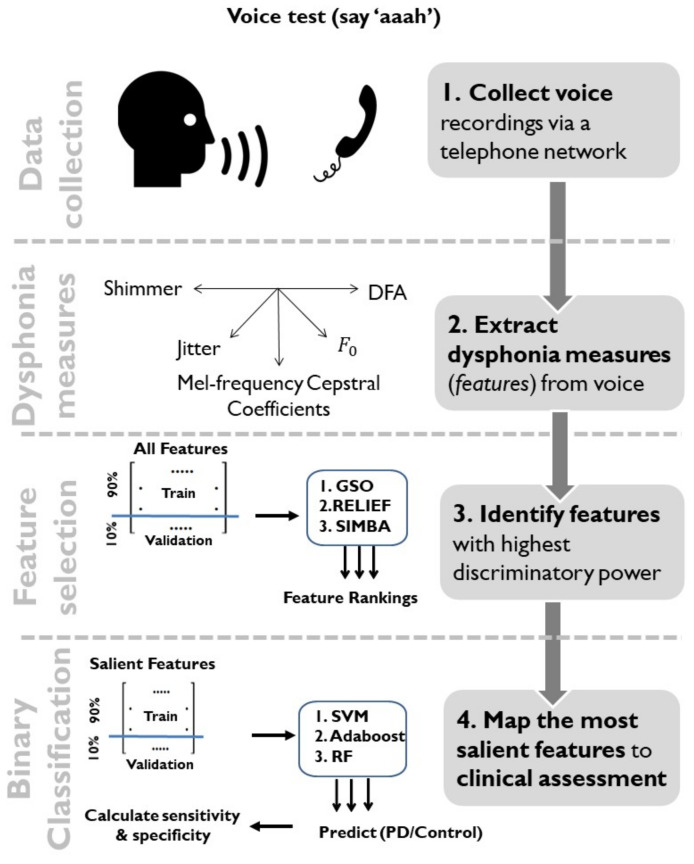
Schematic diagram showing the different stages of this study. Specifically: (**Step 1**) data collection: sustained phonations were collected over a standard telephone line network; (**Step 2**) feature extraction: 304 dysphonia measures were extracted from each phonation to characterize voice impairment; (**Step 3**) feature selection: using a balanced dataset (*n* = 1500 PwP and 1500 control participants), the feature matrix was split into non-overlapping training and test data using a 10-fold cross-validation scheme and three feature selection techniques (GSO, RELIEF, and SIMBA) were employed for identifying the most salient features on the training data; (**Step 4**) classification: the most salient subset of features were mapped onto clinical assessment (PD/Control) using binary classifiers (SVMs, Adaboost, and Random Forests). The final classification step was on the test data held out as part of the CV; subsequently, once we decided on the final statistical learning model, the trained classifier was also presented with the held-out dataset (8942 samples) as an additional performance assessment approach.

Throughout this study we report performance in terms of the accuracy, along with sensitivity and specificity. In the final model validation step, we provide the full confusion matrix to facilitate understanding of the classifier’s output. The full methodology of the study is concisely summarized in Figure 1.

## 3. Results

**Figure 2:**
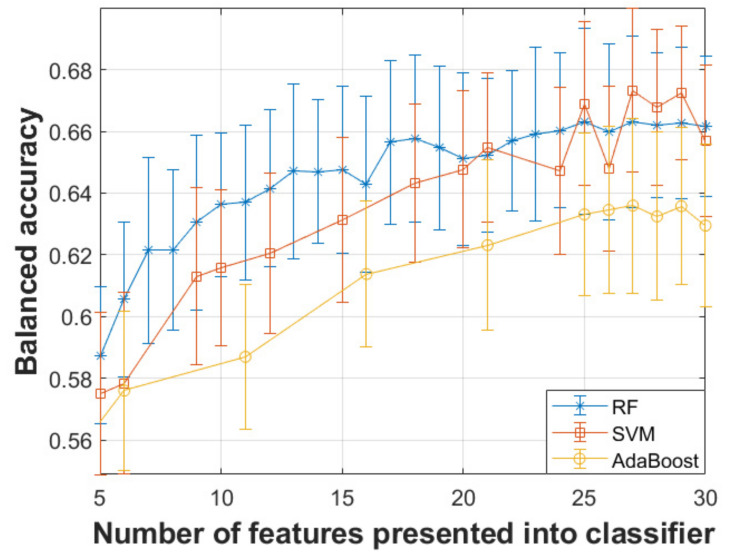
Balanced accuracy as a function of the number of features presented into the three binary classifiers for the validation dataset comprising 3000 samples (1500 controls and 1500 PwP). The bars denote the standard deviation around the quoted mean score. The features presented into the classifiers were selected using SIMBA.

**Figure 3:**
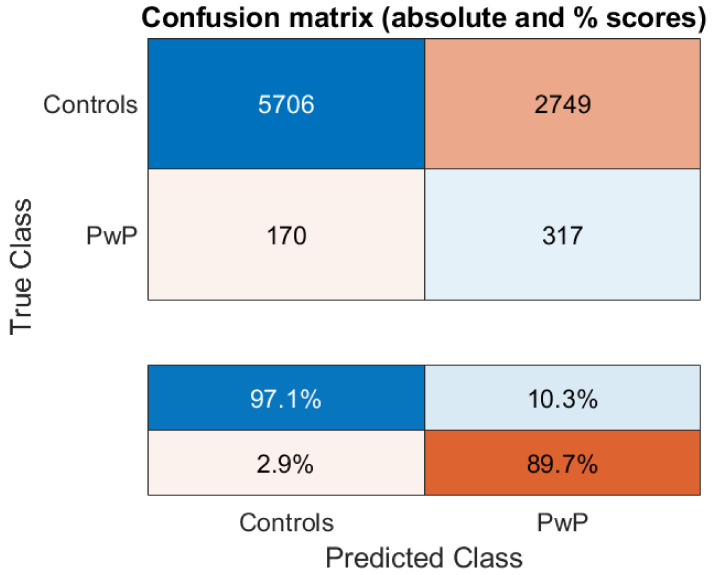
Confusion matrix denoting performance on the held-out unbalanced dataset (*n* = 8942 samples) when using the best performing model selected from the results presented in Figure 2 (SVM with 27 features selected using SIMBA).

Figure 2 illustrates the performance (balanced accuracy) of the model as a function of the features presented into SVM in the standard 10-fold CV setup. Table 3 summarizes the different performance measures of the three classifiers considered in this study for completeness. The performance was evaluated using only the test data, using a 10-fold CV scheme with 100 iterations. We remark that 27 features with an SVM led to a balanced accuracy of about 67.3% (sensitivity: 67.43%, specificity: 67.25%). Therefore, we selected this trained model with the 27 features to test further how well findings generalize on the out-of-sample (held-out) unbalanced dataset. The resulting confusion matrix for the unbalanced held-out dataset (*n* = 8942 samples) is provided in Figure 3 (balanced accuracy: 66.3%).

The results in Figure 3 suggest that we can indeed correctly identify the vast majority of PwP in the held-out (unbalanced) dataset, and this supports the presented methodology as a potentially useful biomarker that could be further explored.

## 4. Discussion 

We investigated the potential of differentiating between PwP and controls using telephone-recorded speech collected under acoustically non-controlled conditions utilizing different statistical machine learning techniques and strategies. This study is part of our wider goal to explore whether we can develop a PD screening tool that is readily accessible, accurate, and ideally free-of-charge, and is the underlying reason we set up the PVI study from which the data for this study were drawn. We demonstrated 67.34% balanced accuracy using 27 acoustic features presented into an SVM with a standard 10-fold CV approach. This finding was further verified on an additional out-of-sample unbalanced dataset where we found a balanced accuracy of 66.3% (sensitivity: 65.09%, specificity: 67.49%). Overall, this is very similar performance to what we had previously reported in Arora et al. (2019) (66.4% balanced accuracy); however, this has now been achieved using 27 acoustic features compared to the 100 features that we had reported in the afore-mentioned study, and so is a more parsimonious result.

Unlike our previous exploration of the ability of the PVI dataset to differentiate PwP from controls, here we used only the US cohort. This was motivated by findings in some of our earlier investigations that some of the feature distributions are different across the PVI cohorts [22], which suggests that we should carefully consider stratifying the PVI data and investigating cohorts independently. We aim to explore transfer learning approaches [40] to account for covariate shifting between the different datasets in the PVI study (given data has been collected across 7 countries and participants between countries may come from different linguistic backgrounds e.g., English or Spanish).

Placing the results in the wider context in the research literature, this study’s findings are very modest given we had previously reported more than 98% binary differentiation between PwP and controls using a similar protocol to collect sustained vowel /a/ phonations [10]. Similarly, other research groups had indicatively reported accuracies around and over 90% in this binary differentiation application [8,41]. However, we stress that previous work had focused on collecting data under carefully controlled acoustic conditions (e.g., sound-treated booths, using high-quality standardized microphones [10,15]), whereas in the PVI participants self-enrolled using their own devices, which have different specifications in terms of microphone quality and frequency attenuation characteristics, and in their own environments, which typically had some background noise, whilst using different telephone networks. Moreover, unlike most research studies, participants in the PVI were not screened or clinically assessed for study enrollment, and thus we cannot rule out the presence of clinical-pathologic differences in voice within this cohort. Collectively, all these ‘degrees of freedom’ lead to lower quality data and therefore it is expected that there will be considerable performance degradation. For example, some of the most successful nonlinear dysphonia measures in this application rely on the use of high frequencies (2.5–10 KHz) to compute the ‘noise’ component in the recorded signal (see [10] for details). Given that the sampling rate in PVI is 8 kHz (and therefore the useful recorded information is up to 4 kHz according to the Nyquist sampling theorem), this constrains the extraction of clinically informative features. 

Speech impairment is commonly associated with Parkinson’s [40] and is characterized by pitch monotonicity, variable rate, imprecise consonants, and breathiness and harshness. As opposed to other types of speech signals that are often used in clinical assessments, such as running speech and reading aloud a linguistically rich pre-specified text e.g., the Grandfather Passage [20], the use of sustained phonations helps circumvent challenges associated with different accents and linguistic confounds [20]. For example, our previous work has shown that sustained phonations can provide high accuracy in differentiating PwP from controls [10], along with other interesting insights in the speech-PD literature, including replicating PD symptom severity and assisting PD rehabilitation [10,12,18,21]. We emphasize also that the methodology adopted in this study for processing sustained vowels had previously also been generalized to analyze different types of speech, e.g., voice fillers [42], and to provide useful insights more widely in different biomedical speech signal processing applications [43]. Therefore, the use of sustained vowels is strongly motivated and has been practically vindicated. A further practical consideration is that this study draws data from PVI, where data were collected across 7 countries with participants coming from different linguistic backgrounds [19]. One of the aims of PVI was to provide cross-linguistic comparisons for the assessment of PD within a short time span of speech samples from a large, self-selected population group. Therefore, for practical reasons and to minimize participant burden, we had decided in PVI to collect exclusively sustained vowels. It is due to these reasons that the focus of this study was on analyzing sustained phonations. Nevertheless, we remark that the use of alternative speech types, e.g., running speech, might be accommodating additional acoustic information which is not captured in sustained vowels (although we stress that the argument goes both ways, the use of sustained vowels may capture information not accounted for in running speech). An interesting line of future work would be to evaluate the efficacy of telephone-quality sustained phonations in conjunction with running speech to develop screening tools for PD.

The participants in this study were entirely self-selected, where they were prompted to answer the question—‘Do you have Parkinson’s disease?’ and their response was treated as the gold standard (or label) for statistical mapping. In the absence of detailed clinical assessments, we cannot rule out clinical-pathologic differences in voice within this cohort, which could be one of the factors contributing to the relatively low discrimination accuracy reported in this study. It is worth noting that diagnosis/monitoring of PD requires in-person subjective assessment, typically by a trained neurologist, which can incur substantial logistical costs in resource-constrained and remote settings. Thus, we deemed it necessary to include only self-reported symptoms. Specifically, the data collection protocol of PVI was designed with the objective to develop a population-based screening (and not monitoring) tool for PD, which would have the potential to transform current practices by reducing logistical costs associated with in-person clinical assessments, while exploring alternate routes to recruiting participants for clinical trials.

This study builds on our previous work on PVI [19] and acoustic analysis [10,12,14,21] to almost completely automate the data processing pipeline. In principle, it may be useful to apply auditory-perceptual analysis relying on human expertise to analyze the data and potentially identify problems, e.g., highly aperiodic/too noisy signals, and also to perceptually characterize the signals (producing additional features). This is indeed often done in studies with a low number of speech samples with speech signals of different nature (e.g., running speech, counting days, reading pre-specified linguistically rich text etc.). Auditory-perceptual analysis is not commonly used when processing sustained vowels, at least in the biomedical speech signal processing literature. Moreover, auditory-perceptual analysis would be very challenging practically and costly for the size of the available data in PVI. Instead, developing automated pattern recognition tools combined with statistical machine learning offers a replicable, objective, automated, and directly scalable approach. This has enabled us to automatically determine, for example, highly aperiodic and noisy signals which were discarded from further analysis (for details on the algorithm see our previous work [19]).

We explored three different feature selection methods and standard feature transformation using PCA to reduce the dimensionality of the dataset. The transformed features using PCA led to consistently worse results and hence these results are not presented in the paper due to space constraints. The three feature selection algorithms led to quite different feature subsets (results not shown), and SIMBA along with SVM provided a somewhat better overall performance in the balanced dataset where we applied the standard CV approach. Therefore, we reported in Figure 2 the performance of classifiers as a function of the number of features progressively selected by SIMBA.

SVMs and RF worked considerably better than Adaboost in this application (see Figure 2). In our experience on this and related PD problems using classification tools, we have observed that generally bagging approaches tend to outperform boosting approaches, although we do not have a theoretical justification for this finding. SVMs led to the best overall result, which is broadly in agreement with our empirical observation in related studies on Parkinson’s applications; we have previously reported SVMs slightly outperform RF in binary classification problems, whereas RF generally leads to better outcomes in multiclass classification problems [21]. Again, this should be cautiously considered on the basis of our experience in related applications, and we make no further claims on generalizability of this finding. We remark that the choice of the three classifiers used here is indicative of some commonly used methods, there are many alternative classifiers that could be explored. For example, an interesting line of further research work would be to provide a comparison of different classification methods, including deep learning. Moreover, it would be worth exploring different classifiers in further detail in conjunction with different class balancing schemes and model validation strategies. 

There are different model validation strategies that could be explored and here it is particularly important because of the highly unbalanced nature of the dataset. In principle, when using a single dataset it is useful to perform CV (e.g., 5-fold or 10-fold CV, along with additional iterations for statistical confidence) rather than leaving a single portion of the data out for testing (‘the testing dataset’). This is because often we want to assess the model’s robustness with perturbed training/test data, while also assessing variability in performance across folds (and iterations) to provide an estimate of the generalization performance including a confidence interval. However, the highly unbalanced nature of the problem given the available dataset in this study poses considerable challenges when using a standard CV approach. Therefore, we decided on a strategy where we used both model validation approaches, retaining a completely separate subset of the data for testing at the very end and using a balanced subset with 3000 randomly selected samples (which overcomes problems with highly unbalanced data) for a standard training/testing scenario using 10-fold CV. This enables us to both assess the model’s performance in a ‘classifier-friendly’ binary classification setting with a balanced dataset where we can also provide a confidence interval on the estimates (see Figure 2) and also to test the model’s performance on an additional unbalanced subset (see Figure 3).

We remark that the developed SVM model was further validated on an unbalanced ‘held-out’ dataset (see Figure 3), where we observe that most PwP were correctly detected. The false positives rate is still fairly high and there is ample space for improving these results further before they can be meaningfully used as an accurate clinical decision support tool. Nonetheless, the findings in Figure 3 highlight that this freely accessible tool for screening for PD might be a useful direction and could be complemented with additional modalities (e.g., smell [44] and smartphone-based tests [11,16,18]) to form a more accurate and practical tool that people could periodically use for mobile check-up and potentially facilitate referrals for specialized physical neurological assessment.

This study has some key limitations primarily regarding the quality of the speech dataset. The standard recommendation of the speech community is that speech signals should be sampled with at least 20 KHz sampling frequency for clinical applications because there is useful information in the higher frequencies of the spectrum [20]. Also, the data in PVI was collected under acoustically non-controlled conditions, which has a clear degradation effect on the data quality of the recorded speech signals. Nevertheless, some recent exploratory work has demonstrated that sustained vowel /a/ transmitted over the simulated standard telephone network (following the typical digital communications process with down-sampling to 8 KHz, encoding, transmitting through a noisy channel and decoding) demonstrated that the reduction in voice quality was not prohibitive for replicating the standard PD symptom severity metric [14]. Therefore, there is some justification that the reduced sampling rate used in PVI (8 KHz) would still be useful information to be extracted from the sub-optimally recorded data. In principle, a study could be designed these days where people could collect speech samples recorded on a high-end smartphone (which uses a high-quality microphone) and captured using a dedicated smartphone app at the recommended sample rate. However, that would require people to have access to high-end expensive equipment, and thus such a solution would not be widely available. Instead, PVI was conceptualized as an approach to democratize access to a potentially useful PD screening tool that could be accessible to all at practically no cost. We maintain that if we want to scale up work and deliver responsible, innovative solutions to make a meaningful differences in practice with a largely accessible tool, there are some compromises we will likely need to make when collecting data in a practical setting so that it would be as accessible as possible by those who would like to use it.

## 5. Conclusions

This study further supports the concept of exploring telephone-quality speech towards developing a screening tool to assess PD with an easy-to-use test relying solely on the use of the sustained vowel /a/. Our findings have important implications toward democratizing access to a useful, generalizable, and robust PD tool at practically no cost, which can be easily used remotely and at scale with any telephone device. This study is a part of the broader work that members of the research community are increasingly focusing, which is developing diagnostic decision support tools in PD which can be adopted at scale. In time, this approach could be expanded to facilitate early diagnosis both for PD and potentially other related conditions. We envisage this tool may be widely applied to provide early probabilistic indication of PD particularly for groups that are at risk, potentially facilitating early PD diagnosis which in turn can lead to better longitudinal symptom management.

## Figures and Tables

**Table 1:** Data details and participant characteristics.

Characteristics	PD Participants	Controls
Number of phonations	1987	9955
Number of participants	1078	5453
Age (years)	62.65 (12.03)	49.19 (15.89)
Male/Female	566/512	2976/2477

Note: Age is reported as mean and standard deviation (in brackets).

**Table 2:** Breakdown of the dysphonia measures used in the study.

Family of Acoustic Measures	Brief Description	Number of Measures
Jitter variants	F0 perturbation	28
Shimmer variants	Amplitude perturbation	21
Harmonics to Noise Ratio (HNR) and Noise to Harmonics Ratio (NHR)	Signal-to-noise, and noise-to-signal ratios computed using standard approaches relying on autocorrelation	4
Glottis Quotient (GQ)	Vocal fold cycle duration changes	3
Glottal to Noise Excitation (GNE)	Extent of noise in speech using energy and nonlinear energy concepts	6
Vocal Fold Excitation Ratio (VFER)	Extent of noise in speech using energy, nonlinear energy, and entropy concepts	9
Empirical Mode Decomposition Excitation Ratio (EMD-ER)	Signal-to-noise ratios using EMD-based energy, nonlinear energy, and entropy	6
Mel Frequency Cepstral Coefficients (MFCC)	Amplitude and spectral fluctuations on the Mel scale quantifying envelope and high frequency aspects	39
F0 related	Comparisons of F0 against age and gender matched controls, including probabilistic variabilities	3
Wavelet-based coefficients	Amplitude, scale, and envelope fluctuations quantified using wavelet coefficients	182
Pitch Period Entropy (PPE)	Variability of F0 expressing inefficiency of F0 stability over and above controls	1
Detrended Fluctuation Analysis (DFA)	Stochastic self-similarity of turbulent noise	1
Recurrence Period Density Entropy (RPDE)	Uncertainty in estimation of F0	1

Algorithmic expressions for the dysphonia measures summarized above are described in detail in [12,21,24,25]. The MATLAB source code for the computation of the dysphonia measures is freely available from the last author’s group website: https://www.darth-group.com/software (last accessed 10 October 2021). F0 refers to fundamental frequency estimates, here computed using SWIPE [28].

**Table 3:** Out-of-sample performance measures for the three classifiers (SVM, Adaboost, and Random Forests) using 10-fold CV with 100 iterations on the balanced dataset (*n* = 3000 samples).

Classifier	Number of Optimal Features	Sensitivity	Specificity	Balanced Accuracy
SVM	27	**67.43%**	**67.25%**	67.34%
Random Forests	27	66.38%	66.20%	66.29%
Adaboost	27	63.11%	63.60%	63.36%

Note: highest scores are highlighted in bold.

## Data Availability

Due to data confidentiality, the data cannot be made publicly available. Interested researchers seeking to explore collaborative opportunities can approach the authors.
